# The research status and prospect of Periostin in chronic kidney disease

**DOI:** 10.1080/0886022X.2020.1846562

**Published:** 2020-11-26

**Authors:** Yuan-yuan Jia, Yue Yu, Hong -jun Li

**Affiliations:** a Department of Health Management Medical Center, The Third Hospital of Jilin University, Changchun, China; b Department of Endocrinology and Metabolism, The Third Hospital of Jilin University, Changchun, China

**Keywords:** Chronic kidney disease (CKD), diagnosis, extracellular matrix(ECM), periostin, therapeutic target

## Abstract

The continuous accumulation of extracellular matrix will eventually lead to glomerular sclerosis, interstitial fibrosis, tubular atrophy and vascular sclerosis, which are involved in the progression of chronic kidney disease (CKD). If these processes can be discovered early and effective interventions given in time, the progression of kidney disease may be delayed. Therefore, exploring new biomarkers and therapeutic targets that can identify CKD at an early stage is urgently needed. In recent years, studies have shown that urine periostin may be used as a marker of early renal tubular injury. And in an animal model experiment of hypertensive nephropathy, periostin is involved in the progression of kidney injury and reflects its progression. Here we review the current progress on the role of periostin in pathologic pathways of kidney system to explore whether periostin is a potential therapeutic target for the treatment of CKD.

## 1. Introduction: chronic kidney disease

Chronic kidney disease (CKD) refers to the irreversible changes of renal structure and function caused by various reasons, which last for months or years. CKD is a disease that threatens public health and is charactered by high prevalence, high disabilities, and high medical expenses. If not treated promptly in the early or middle stage, it will eventually progress to end-stage renal disease (ESRD), which is seriously harmful to human health.

The continuous accumulation of extracellular matrix eventually leads to glomerular sclerosis, interstitial fibrosis, tubular atrophy and vascular sclerosis, which are involved in the progression of CKD [1,2]. If early diagnosis and effective interventions are given in time, the progression of kidney disease may be delayed. Therefore, exploring new biomarkers and therapeutic targets that can identify CKD at an early stage is an urgent problem to be solved. Some current studies suggest that IL-22, TGF- 1, Mir-218, DDR2 and FFNT25 may be biological markers and therapeutic targets for CKD [3–6]. In recent years, Satirapoj et al. [7] found that urine periostin can be used as a marker of early renal tubular injury, and Guerrot et al. [8] found that in an animal model experiment of hypertensive nephropathy, periostin is involved in the progression of renal injury and reflecting its progress.The aim of this review is to summarize recent knowledge about the role of periostin in the pathogenesis of CKD.

## 2. Biological characteristics and functions of Periostin

### 2.1. The structure and expression of Periostin

Periostin contains a typical signal sequence, 4 cysteine-rich repeat domains and a C-terminal variable domain [9,10]. It is structurally homologous to βig-H3 (a 68 kDa transforming growth factor-β1 induced protein) and insect neural cell adhesion protein (fascilin) [10–13]. These proteins are essential for interacting with integrins on the cell surface. The C-terminal region of periostin regulates the composition and interaction between cells and matrix by combining various extracellular matrix proteins such as type I and type V collagen, fibronectin, Tenascin-C, and acid mucopolysaccharide [14]. Early researches suggested that periostin is specifically expressed in periosteal [15] and periodontal ligament tissue [16]. Currently, it has been confirmed that periostin exists in many tissues, such as heart, skin, tumor and blood vessels, with the highest content in the aorta, lower digestive tract, placenta, uterus, thyroid tissue and breast [15,17–21]. However, most studies have shown that periostin expression is significantly increased in different renal pathological tissues, and it is significantly related to the decline in renal function. In addition, the expression of periostin will also be significantly increased in pathological processes such as tumors, myocardial infarction, and wound repair [22].

### 2.2. Physiological function of Periostin

Physiological expression of periostin has important functions. Periostin is involved in maintaining the function of periodontal ligament cells and regulating the formation of periodontal ligament collagen fibers, which can promote the adhesion, proliferation, differentiation, and the periodontal-like and cementoid-like effects of periodontal ligament cells. During the formation of periodontal ligament collagen fibers, periostin can coexist with collagen, and maintain the integrity of the periodontal ligament fiber system under mechanical stress stimulation [23]. Additionally, periostin promotes wound healing. Ontsuka et al. [24] showed that periostin was significantly deposited under the granulation tissue and the dermal-epidermal junction of wound margin. Mice lacking periostin showed delayed would healing, which can be improved after applying exogenous periostin. The proposed mechanism is periostin accelerates skin wound healing by activating fibroblasts. In addition, in cardiology, the expression of periostin is rapidly up-regulated when the myocardium damaged or during cardiac remodeling, which helps the heart to heal and induces the proliferation of differentiated cardiomyocytes, thereby accelerating the repair process of myocardial infarction [8,25–34].

## 3. The role of periostin in CKD

### 3.1. Periostin can be used as a marker to diagnose CKD and reflect the severity of CKD

During the development of the kidney, periostin is highly expressed in the peri-renal interstitium and the periureteral interstitium. After the development completed, the expression of periostin is significantly reduced [35]. In the pathological tissues of CKD, most studies have shown that the expression of periostin is significantly increased, and related to the decline of renal function. Satirapoj et al. [7] reported that the expression of mRNA and protein of periostin in various animal models, such as streptozotocin induced diabetic nephropathy (SZ-DN) and uni-lateral ureteral obstruction (UUO), are incresed, which mainly expressed in the cytoplasm of the renal tubular epithelial cells.

In the kidneys of patients with diabetic nephropathy, periostin is mainly expressed in the atrophied and non-atrophied tubule epithelial cells and the sclerotic glomerular mesangial area. When urine protein has not increased in the early stage of diabetic nephropathy, urine periostin has been significantly increased, which is also related to proteinuria and estimated glomerular filtration rate (eGFR), suggesting periostin may be an early and sensitive marker of renal injury [36]. Periostin is significantly increased in the urine of patients with allograft nephropathy. It is positively correlated with urine protein creatinine ratio and blood creatinine, and negatively correlated with eGFR, suggesting that periostin can be used as a potential biomarker to reflect the severity of CKD [37] (Table 1). The above shows that the detection of urine periostin can be used as an important marker for assisting early diagnosis and evaluating the progress of CKD, which is charactered as noninvasive, rapid, sensitive and repeatable. The specific mechanism of increasing urinary periostin in CKD patients is still unclear. According to the existing research [39], urine periostin is mostly derived from renal tubules. It is speculated that it may be derived from the residual secretion capacity of renal tubular epithelial cells or directly released by exfoliated cells, while it is not ruled out whether it is partially derived from glomerular filtration. Normal glomerular filtration membrane allows proteins with a relative molecular mass below 40 000 to pass smoothly [40]. However, when the molecular barrier of the glomerular filtration membrane is damaged, serum periostin may enter the urine through it. We can further mark the serum periostin to calculate its filtration status. Periostin is highly expressed in urine and pathological tissues of CKD patients. When the myocardium is damaged or the heart is remodeled, the expression of periostin is rapidly increased to help the heart heal, inducing the proliferation of differentiated cardiomyocytes, thereby accelerating the repair process of myocardial infarction. Periostin can also promote the proliferation of inflammatory tissues (such as asthma, rhinitis, etc.). Izuhara et al. [41] showed that relative to other serum proteins, the content of periostin in the serum of healthy adults is low, generally less than 50 μg/L, and its increase will be easier to detect. Kanemitsu et al. [42] found that serum

**Table 1.** Studies on Periostin as a biomarker in CKD.

Disease types	Object of study	Results and conclusions
Diabetic Kidney Disease [7]	Patients	Urine perioetin was increased significantly in patients with diabetic kidney disease, which was positively correlated with urinary protein and negatively correlated with eGFR, suggesting urine Periostin can be used as a marker of early renal tubular injury, and reflect the severity of diabetic kidney disease.
Lupus nephritis [38]	Patients	Periostin staining score of lupus nephritis tissue is correlated with the chronic index (CI) of renal pathology, which is positively correlated with blood creatinine and urea nitrogen and negatively correlated with eGFR, suggesting that Periostin can be used as a marker of renal injury.
Nephrotic syndrome [23]	Patients	Periostin is mainly expressed in the areas of mesangial hyperplasia and interstitial fibrosis. Periostin mRNA is significantly increased in the pathological tissues of lupus nephritis and focal segmental glomerulosclerosis. Besides, it has an increasing trend in minimal change nephrotic syndrome and membranous nephropathy and IgA nephropathy, and the difference is not statistically significant. Periosin mRNA is negatively correlated with eGFR in patients with nephrotic syndrome, and has no correlation with proteinuria and age, indicating that it can be used as a biomarker.
Hypertensive nephropathy [8]	Mice	The mRNA expression of periostin is increased in hypertensive nephropathy tissues, and is closely related to blood creatinine, urine protein and renal blood flow, suggesting that periosin may reflect the severity of hypertensive nephropathy. After effective treatment, the protein expression of periosin in renal tissue is significantly reduced, and the contents of ET4 and E-selectin, the markers of the vascular endothelial dysfunction, have little change compared with that before, suggesting that periostin are more sensitive in reflecting the outcome of renal injury in hypertension.
chronic allograft nephropathy [37]	Mic	The expression of urine periostin in patients with chronic transplant kidney nephropathy is increased, which is positively correlated with urine protein creatinine ratio and blood creatinine, and negatively correlated with eGFR.Its sensitivity and specificity for the diagnosis of CKD are high.

Abbreviations: CKD: chronic kidney disease; eGFR: estimated glomerular filtration rate.

periostin was significantly increased in patients with allergic asthma. Ling et al. [43] reported that high level of serum periostin in patients with acute myocardial infarction (AMI) suggests a poor prognosis. Yamashita et al. [44] found that serum periostin in patients with coronary heart disease was significantly higher than that in healthy adults. Lv et al. [45] demonstrated that preoperative serum periostin can be used as an independent biomarker to reflect the prognosis in liver cancer patients. Due to inflammation, arteriosclerosis, and fibrosis also play an important role in the progression of CKD, we can further study the expression of serum periostin in CKD patients.

### 3.2. Assessing the prognosis of CKD based on Periostin

In recent years, many clinical studies have shown that periostin reflects the prognosis of various diseases. Ling et al. [43] demonstrated that AMI patients with high serum levels of periostin had a poor short-term prognosis, and serum periostin level was positively correlated with Killip grade of myocardial infarction(It is divided into 4 grades in total, including grade I:no obvious heart failure; grade II:left heart failure, lung rales < 50% lung field; grade III:acute pulmonary edema; grade IV:cardiogenic shock, There are different stages and degrees of hemodynamic changes.), and negatively correlated with left ventricular ejection fraction and left atrium diameter, suggesting that serum periostin levels can be used as a predictor of short-term poor prognosis in AMI patients(all types of myocardial infarction patients). Additionally, some studies showed that periostin is closely related to the prognosis of many tumors (such as breast cancer [46], non-small cell lung cancer [47], prostate cancer [48], etc.). Regarding periostin’s role in the prognosis of renal disease, Sen et al. [49] found only in the mouse model of hypertensive nephropathy that the level of periostin mRNA in renal tissue that achieved clinical remission after effective treatment decreased significantly. The level of periostin mRNA was positively correlated with blood creatinine and urine protein levels, and negatively correlated with renal blood flow, suggesting that periostin may have the potential to reflect the outcome, prognosis and efficacy prediction of renal disease. However, there has been no clinical research in this area, and further studies on the expressions of serum and urine periostin are needed to predict whether it can reflect the progress of the disease and predict its appropriate level when treating patients with kidney disease.

## 4. Research progress on the mechanism of Perisotin in kidney disease

### 4.1. Periostin binds to integrin receptor

A study found that [26] periostin and integrin share the spatial expression of αβ subunits, and the expression of periostin and αβ integrin can be detected in glomerular mesangial cells. Periostin can activate integrin-linked kinase(ILK) and affect the survival, proliferation, differentiation and apoptosis of cells by binding to integrin α/β subunits, participating in the progression of glomerular diseases accompanied with proteinuria [29,30,50]. Periostin can also participate in the proliferation of renal cyst cells through the Periostin-ILK-AKT-mTOR signal axis [51].

### 4.2. Periostin is involved in Ang II-mediated fibrosis pathway

The renin-angiotensin system is a core member of multiple mechanisms leading to the progression of renal fibrosis. Studies have shown that Ang II can induce the expression of periostin in fibroblasts and vascular smooth muscle cells through Ras/p38MAPK, CREB, ERK1/2, TGF-β1 pathway and PI3K signaling pathway [52]. Guerrot et al. [8] found that in a mouse model of hypertensive nephropathy, periostin was mainly stained in the damaged area of the kidney. In immunohistochemically analysis, it was found that periostin was mainly located around blood vessels. After blocking the effect of Ang II, it can improve renal hemodynamics, reduce proteinuria and the expression of periostin in renal tissue, suggesting that periostin may be involved in the process of Ang II-induced renal fibrosis, but its specific mechanism remains to be studied.

### 4.3. TGF-β mediates the pathway of renal fibrosis

It has been reported that epithelial-mesenchymal transition(EMT) can lead to tubulointerstitial fibrosis and the progression of diabetic nephropathy.In recent years, studies have found that TGF-β promotes the transformation of renal epithelial tissue to mesenchymal tissue in patients with diabetic nephropathy [53–56]. It is speculated that the role of periostin in kidney injury and renal tissue remodeling is similar to that of other tissue injuries [53–56]. Periostin can induce cell dedifferentiation, increase TGF-β expression and extracellular matrix deposition. In addition, TGF-β can also promote the expression of periostin, which further promotes the loss of renal tubular epithelial phenotype and ultimately leads to fibrosis [12]. The specific mechanism may be that TGF-β regulates expression levels of a series of miRNA(miR) through the intracellular signal transduction protein Smad3, which ultimately leads to renal fibrosis [57].

## 5. Periostin as a marker for monitoring the disease condition and diagnosis of Kidney disease

### 5.1. Periostin and glomerular disease

It is reported that the expression of glomerular periostin mRNA is highly upregulated in different nephrotic patients. the study found that the expressions of periostin mRNA are significantly increased in pathological tissues such as focal segmental glomerulosclerosis, membranous nephropathy,lupus nephritis, etc. eGFR is negatively correlated to the transcription level of periostin.By immunohistochemistry, it was found that periostin is mainly expressed in the areas of mesangial hyperplasia and interstitial fibrosis, and the expression level is negatively correlated with renal function [38]. Subsequent studies related to lupus nephritis found that the periostin staining score of lupus nephritis tissue is correlated with the chronic index (CI) of renal pathology, and is positively correlated with blood creatinine and urea nitrogen and negatively correlated with eGFR. These studies suggest that periostin has the potential to monitor the progression of kidney disease.

### 5.2. Periostin and diabetic nephropathy

Studies have shown that periostin is mainly produced by distal tubules, and the interstitial transformation tendency of distal tubules in various kidney injuries can be reflected by periostin [39]. Another study suggests that renal tubular injury may precede glomerular injury of diabetic nephropathy [50], which may be the reason for the excretion of several urinary biomarkers occurs earlier than albumin. Therefore, a new urine biomarker for early diagnosis of diabetic nephropathy is gradually proposed. Recently, a clinical study [7] evaluated the clinical significance of urine periostin to patients with type 2 diabetic nephropathy. The study included 30 healthy volunteers and 328 type 2 diabetic nephropathy patients with normal proteinuria (*n* = 114), trace proteinuria (*n* = 100) or massive proteinuria (*n* = 114), using an enzyme-linked immunosorbent assay to determine urine periostin content. The results showed that the urine periostin levels of patients in the normal proteinuria group, the trace proteinuria group, and the massive proteinuria group were significantly higher than the normal control group. The increase of urine periostin can be detected before trace albuminuria. This suggests that periostin may be a biomarker for early kidney damage in type 2 diabetic nephropathy, and measuring urine periostin in patients with type 2 diabetes may help to provide an early diagnosis and advanced interventions.

## 6. Conclusion and perspective

Periostin is a versatile cell matrix protein whose expression is increased during tissue mechanical stress or injury, with physiological functions related to the improvement of many tissue injuries repair. In recent years, periostin has received increasing attention in the field of kidney disease. Literatures show that periostin is closely related to various kidney diseases and their progression, fibrosis and prognosis. Increasing evidence shows that periostin may be used as a new diagnostic marker and therapeutic target for various kidney diseases, and the relevance of its clinical practice should receive more important research. However, at present, there are relatively few clinical researches on periostin in the field of kidney disease worldwide. It is feasible to conduct prospective clinical research on periostin, and it is of great clinical significance to develop new diagnostic methods by using periostin. The specific pathogenic mechanism of periostin in many diseases, especially kidney disease, is yet unclear, and further exploration on its specific signaling pathways are needed to find new therapeutic targets. According to the current researches, periostin can be used as a biological marker for early detection of disease and clinical diagnosis, a potential therapeutic target and a prognostic indicator for various diseases. Recently, studies on periostin in kidney disease also have demonstrated the above effects, while most of them are conducted on animals. Especially, there is no relevant clinical research at present on reflecting the prognosis of CKD worldwide. More clinical studies are needed to observe the changes of periostin in patients’ serum and urine, and to clarify its role in CKD outcome and prognosis.
